# Neural Plasticity during Aging

**DOI:** 10.1155/2019/6042132

**Published:** 2019-03-26

**Authors:** Mauricio Arcos-Burgos, Francisco Lopera, Diego Sepulveda-Falla, Claudio Mastronardi

**Affiliations:** ^1^Institute of Translational Medicine, Universidad del Rosario, Bogotá, Colombia; ^2^INPAC Research Group, Fundación Universitaria Sanitas, Bogotá, Colombia; ^3^Neuroscience Research Group, University of Antioquia, Medellín, Colombia; ^4^Institute of Neuropathology, University Medical Center Hamburg-Eppendorf, Hamburg, Germany

The percentage of older people is increasing steadily in the proportion of the total population of the world. In a recent report published by the National Institute on Aging, in March of 2016, it was estimated that 8.5 percent of people, globally accounting for nearly 617 million, are aged 65 and over [1]. Additionally, it is also predicted that this ratio is going to double, reaching 17 percent of the global population (1.6 billion elders) in 2050 [1]. Consequently, the incidence of neurodegenerative diseases will continue to rise as a result of the increase in life expectancy.

Emerging evidence suggests that mental health decline due to neurodegenerative conditions constitutes the largest cause of global disability, which is accountable for over 20% of lifespan [2]. Data arising from family studies that compared the age of death of monozygotic and dizygotic twins suggested that approximately 25% of the variation in human longevity could be due to genetic factors [3]. It is noteworthy to remark that this genetic component appears to have a larger impact at older ages. However, only a few genes have been so far associated with lifespan, and the interaction among these genes, epigenetic factors, and environmental regulators are far from being well understood.

The neural plasticity processes occurring during aging are astonishing. For instance, there is mounting evidence supporting the concept that development, ageing, and brain degeneration are not mutually exclusive. It is now quite clear that once the brain is fully developed, it gradually shrinks at different levels during the ageing process [4]. All aged brains exhibit small distinctive alterations linked to neurodegeneration, namely, progressive loss of structure, function, or number of neurons [5, 6]. Neurodegeneration is, to some extent, a natural process occurring towards the end of life [7]. However, it is noteworthy to remark that developmentally related processes, i.e., neurogenesis, can also occur within the adult and ageing brain. In fact, approximately two decades ago, it became evident that a new type of neuroplasticity, one that is related to the addition of new neurons, occurs in the human brain [8, 9]. One of the key brain areas where adult neurogenesis occurs during the human's lifetime is the dentate gyrus, a region of the hippocampus that is essential for memory encoding [8, 10, 11]. There are a number of factors that can alter all of the neural plasticity changes that occur during aging. The understanding of the impact they exert particularly on the brain, and generally on the body, may aid in the development of novel therapeutic strategies to overcome the deleterious effects occurring during the aging process.

Therefore, there is an increased need to expand the knowledge on the different genetic, epigenetic, and molecular pathways and environmental factors that affect brain plasticity and healthy aging. Some of the topics covered in this special issue include the following:

- Environmental factors affecting healthy aging: possible impact of exercise and proton pump inhibitors (PPI)
- Genetic structural variants associated with healthy aging
- The updating of imaging studies to assess neural plasticity during aging
- Possible novel brain areas undergoing neural plasticity
- Preclinical models of healthy aging: protective role of environmental enrichment preventing declarative learning and memory decline

Fourteen papers were submitted for this special issue. Our distinguished reviewers from respective research fields narrowed the field to seven papers, which were finally accepted. The following is a short summary of the findings of each of these papers.

Debate continues in regard to the possible impact of the use of proton pump inhibitors (PPI) and its consequences and links to dementia including Alzheimer's disease. G. Ortiz-Guerrero et al. reviewed preclinical and clinical studies regarding the action of PPI on the central nervous system and their possible implication in the pathophysiology of dementia including Alzheimer's disease. The authors summarized a comprehensive amount of data from a neurobiological and clinical perspective. They discussed both possible neurotoxic and antineurotoxic actions of PPI. They concluded that there is no real consensus on the role of PPIs and the associated risk of dementia. Nevertheless, they gave a word of caution stating that “nutritional and electrolyte monitoring is required in patients who chronically use PPIs, mainly older adults and patients with chronic malnutrition or debilitating chronic conditions”.

With more than 350 million sufferers, depression continues to be the most prevalent mental health problem that largely affects the elderly population worldwide. X. Liu et al. designed a neuroimaging study that could be useful to predict the impact of major depressive disorders on dementia. They used resting-state functional magnetic resonance imaging to study 16 patients who had mild cognitive impairment (MCI) with depressive symptoms and 18 patients with nondepressed MCI. The authors studied their brains to measure the amplitude and synchronization of low-frequency brain fluctuations (ALFF), functional connectivity density (FCD), and coupling, which were quantified as the correlations between ALFF values and their associated FCDs. The authors described specific differences between the two experimental groups in some of these parameters in brain areas such as the medial prefrontal cortex, right precentral gyrus, and medial temporal gyrus that could be relevant to define the impact of depression on the neuropathophysiology of MCI.

Declarative learning involves memorizing concepts and events of our life, which can be expressed explicitly. The decline in memory performance is a distinctive feature of the normal aging process. In their original mouse study, S. Cintoli et al. described for the first time the protective role of environmental enrichment in preventing declarative learning and memory decline in aged mice. Thus, their exciting results in mice suggest that exposure to stimulating environmental conditions could be used as a powerful paradigm to promote better memory performance during aging in the elderly population.

Genetics plays an essential role in the aging process. Interestingly, the genome of neuronal cells displays genomic mosaicism, which includes DNA copy number variations (CNVs). D. Villela et al. investigated for the first time the features of somatic CNV mosaicism in nondiseased elderly brains. In their original study, the authors demonstrated a highly significant increase in the number of CNVs in two brain areas (frontal cortex and cerebellum) when compared with paired blood samples (same individuals). It is noteworthy to remark that almost all evidence of genome structural variation in human brains is derived from studies describing changes in single cells, which were interpreted as originating from independent, isolated mutational events. In their studies, D. Vilella et al. indicated the occurrence of extensive clonal mosaicism of CNVs within the human brain, which reveals a novel type of variation that had not been previously characterized.

Even though a decline in motor function is a common phenomenon that takes place during aging, the functional changes occurring in neural networks responsible for generating movement are far from being understood. Recordings from the primary motor cortex during periods of steady muscle contraction show oscillatory neural activity that is coherent with the activity of contralateral muscles. M. E. Spedden et al. investigated the functional oscillatory coupling between activities in the sensorimotor cortex and ankle muscles during static contraction in fifteen young and fifteen older subjects. Their results show that there is an age-related decrease in the strength of oscillatory corticospinal activity during steady-state motor output. They also conclude that their novel findings might be instrumental in developing new preventive and therapeutic interventions that may strengthen sensorimotor control in elderly subjects.

Mounting evidence suggests that exercise causes beneficial effects on neural plasticity and cognition. Exercise triggers the release of neurotrophins, which can increase neurogenesis, synaptogenesis, and angiogenesis. Additionally, exercise can induce neuroendocrinological changes, which impact positively on cognitive, affective, and behavioral functioning. Even though motor performance declines during aging, learning capabilities remain intact. In their original paper, L. Hübner et al. (in press) reveal for the first time that acute exercise facilitates fine motor control performance and learning, as well as electrophysiological processing in healthy older adults. The authors suggest that their findings could be translated into practice by implementing acute exercise as a method to create successful experiences in fine motor control performance, which ultimately could contribute in motivating older patients' rehabilitation process (Lena Hübner et al. in press).

There are an estimated 47 million people worldwide that suffer from dementia, a number that is projected to increase to approximately 131 million by 2050. Alzheimer's disease is the most common type of dementia accounting for 60-70% of all of the dementia cases. Novel biomarkers for prediction, diagnosis, and follow-up of this neurodegenerative condition are very much needed. Interestingly, functional and pathological mechanisms of the visual system share some similarities with the CNS. In their comprehensive review “Visual Features in Alzheimer's Disease: From Basic Mechanisms to Clinical Overview,” the authors summarize the current evidence describing the pathophysiological alterations occurring in the vision system of patients suffering Alzheimer's disease. The authors foresee that novel objective measurement of vascular and inflammatory changes in the eye may play an essential role in the evaluation of early stages of dementia and AD.

We believe that this special issue would provide new insights into important aspects of neural plasticity-associated aging. The different research perspectives described in this special issue might encourage the undertaking of novel projects aimed at developing novel therapeutic strategies and scientific approaches to tackle some of the health issues that are aggravated by the aging process.

Finally, we would like to give thanks to all of the authors, the reviewers, the editorial board members, and the general staff of Neural Plasticity for their concerted effort in constructing this special issue.
